# Converging Intracranial Markers of Conscious Access

**DOI:** 10.1371/journal.pbio.1000061

**Published:** 2009-03-17

**Authors:** Raphaël Gaillard, Stanislas Dehaene, Claude Adam, Stéphane Clémenceau, Dominique Hasboun, Michel Baulac, Laurent Cohen, Lionel Naccache

**Affiliations:** 1 INSERM, Cognitive Neuro-imaging Unit, Institut Fédératif de Recherche (IFR) 49, Gif sur Yvette, France; 2 Centre Hospitalier Sainte Anne, Service Hospitalo-Universitaire de Santé Mentale et de Thérapeutique, Paris, France; 3 Université Paris Descartes, Paris, France; 4 CEA, I2BM, NeuroSpin center, Gif sur Yvette, France; 5 Collège de France, Paris, France; 6 Assistance Publique Hôpitaux de Paris, Hôpital de la Pitié-Salpêtrière, Pôle des Maladies du Système Nerveux, Paris, France; 7 Université Pierre et Marie Curie Paris 6, Département de Physiologie, Paris, France; National Institutes of Health, United States of America

## Abstract

We compared conscious and nonconscious processing of briefly flashed words using a visual masking procedure while recording intracranial electroencephalogram (iEEG) in ten patients. Nonconscious processing of masked words was observed in multiple cortical areas, mostly within an early time window (<300 ms), accompanied by induced gamma-band activity, but without coherent long-distance neural activity, suggesting a quickly dissipating feedforward wave. In contrast, conscious processing of unmasked words was characterized by the convergence of four distinct neurophysiological markers: sustained voltage changes, particularly in prefrontal cortex, large increases in spectral power in the gamma band, increases in long-distance phase synchrony in the beta range, and increases in long-range Granger causality. We argue that all of those measures provide distinct windows into the same distributed state of conscious processing. These results have a direct impact on current theoretical discussions concerning the neural correlates of conscious access.

## Author Summary

What is the neural signature of the conscious perception of a visual stimulus? To address this question, we recorded neural activity directly from the brains of human subjects (who were undergoing neural surgery for medical reasons). This rare opportunity afforded greater spatial and temporal resolution than noninvasive methods used previously to probe the neural basis of consciousness. We compared neural activity concomitant with conscious and nonconscious processing of words by using a visual masking procedure that allowed us to manipulate the conscious visibility of briefly masked words. Nonconscious processing of words elicited short-lasting activity across multiple cortical areas, including parietal and visual areas. In sharp contrast, only consciously perceived words were accompanied by long-lasting effects (>200 ms) across a great variety of cortical sites, with a special involvement of the prefrontal lobes. This sustained pattern of neural activity was characterized by a specific increase of coherence between distant areas, suggesting conscious perception is broadcasted widely across the cortex.

## Abstract 3

A neural signature of the conscious perception of words by humans is readable in intracranial electrophysiological recordings, which afford relatively high spatial- and temporal-resolution measurements of neural activity.

## Introduction

The neural correlates of consciousness (NCC) still remain highly controversial. Indeed, the precise timing, location, and dynamics of neural events causing conscious access are not clearly and unequivocally determined. Do the NCC correspond to late [1,2] or early brain events [3–10]? Are they systematically associated with reentrant “top down” processing [5,9,11–15]? If so, do they necessarily involve long-range coherent activity [16–21], including prefrontal cortex as an essential node [22–25], or can they be restricted to local patterns of reverberating activity [3–6,8,11,13,15,26–29]? Is the concept of “integrated information” relevant, rather than the specific localization of the underlying cerebral network [21]?

In addition to such fundamental questions, an important methodological issue also remains open. Neural data relevant to conscious access originate from a diversity of techniques including hemodynamic blood oxygen level dependent (BOLD) functional magnetic resonance imaging (fMRI) or positron emission tomography (PET) responses and electrophysiological measures using scalp and intracranial event-related potentials (iERPs), event-related spectral perturbations (ERSPs), and phase synchrony parameters. How are these distinct measures of conscious access related to each other? Do they reflect common facets of the same underlying phenomenon?

In this work, we address some of these issues using intracerebral electrophysiological recordings of neural activity in a group of implanted epileptic patients presented with visually masked and unmasked printed words. This method offers a unique opportunity to measure neural correlates of conscious access with both millisecond time resolution and centimetric spatial resolution. Its high signal-to-noise ratio allowed us to compute several neurophysiological measures from the intracerebral signal in order to unravel the relations prevailing between iERPs, ERSPs, interelectrode phase synchrony, and a recently proposed estimate of causality (Granger causality).

### The Global Workspace Model of Consciousness

We adopted a theory-driven approach, trying to test experimentally a set of explicit predictions derived from the global workspace model of conscious access. This model, in part inspired from Bernard Baars' theory [30], proposes that at any given time, many modular cerebral networks are active in parallel and process information in an unconscious manner [22,23,31,32]. Incoming visual information becomes conscious, however, if and only if the three following conditions are met [23]: Condition 1: information must be explicitly represented by the neuronal firing of perceptual networks located in visual cortical areas coding for the specific features of the conscious percept. Condition 2: this neuronal representation must reach a minimal threshold of duration and intensity necessary for access to a second stage of processing, associated with a distributed cortical network involved in particular parietal and prefrontal cortices. Condition 3: through joint bottom-up propagation and top-down attentional amplification, the ensuing brain-scale neural assembly must “ignite” into a self-sustained reverberant state of coherent activity that involves many neurons distributed throughout the brain.

Why would this ignited state correspond to a conscious state? The key idea behind the workspace model is that because of its massive interconnectivity, the active coherent assembly of workspace neurons can distribute its contents to a great variety of other brain processors, thus making this information globally available. The global workspace model postulates that this global availability of information is what we subjectively experience as a conscious state. Neurophysiological, anatomical, and brain-imaging data strongly argue for a major role of prefrontal cortex, anterior cingulate, and the associative areas that connect to them, in creating the postulated brain-scale workspace.

### Scope and Limits of Our Experimental Paradigm

**Figure 1:**
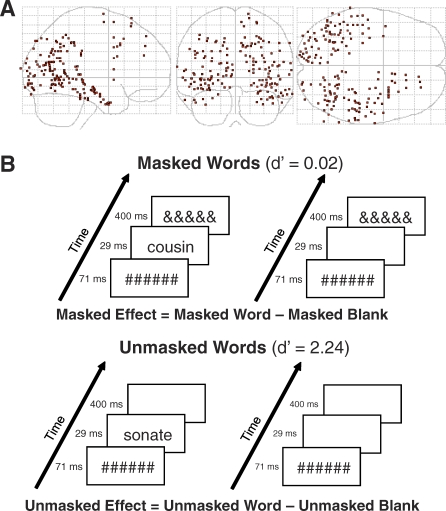
Electrode Locations and Experimental Design (A) Sagittal (left), coronal (middle), and axial (right) normalized glass brain showing all 176 electrodes after normalization in Talairach's anatomical space. (B) Experimental paradigm used to present masked and unmasked words, with *d*′ measures in the forced-choice semantic task.

In the present work, we measured the neural correlates of visually masked words and contrasted them with those of consciously visible unmasked words. On each trial, patients were randomly presented with a masked word, a visible word, or with corresponding control stimuli in which the words were replaced by blank screens. In the masked condition, words or blank screens were presented for 29 ms, preceded by a forward mask and followed by a backward mask. In the unmasked conditions, words or blank screens were made visible by simply removing the backward mask (see Materials and Methods and Figure 1 for details). In order to discard activations induced by the masks, we always subtracted from word-present conditions the corresponding blank condition. This subtraction allowed us to isolate the entire processing path evoked by the masked or unmasked word.

#### Advantages of the visual masking paradigm.

Variants of the masking paradigm have been extensively used in behavioral and brain-imaging studies over the last 30 years. In humans, a cumulative set of data demonstrated that a masked visual stimulus (e.g., word, number, or image) that cannot be reported can nevertheless be processed from low-level visual stages up to abstract cognitive processes, including semantic content, and eventually up to motor response preparation [33]. From a neural point of view, masked stimuli activate a large set of cortical structures, from occipital to anterior frontal regions [34–37]. The interpretation of these observations is informed by recent studies of the mechanisms of visual masking. Recordings of single neurons in nonhuman primates revealed that masking acts by reducing or interrupting the late activity evoked by stimuli while leaving the initial feedforward activation largely unaffected [1,14,27,38–43]. Recent scalp–event-related potential (ERP) studies suggest that the same mechanism may prevail in humans [1,44]. Finally, among the various methods that can be used to compare conscious and nonconscious processes, the masking paradigm allows for a comparison between two very stable and clear-cut states of perception: (1) a strong masking condition in which subjective report and objective discrimination are impossible, and (2) an unmasked condition in which undisputed subjective and objective measures of conscious access can be gathered.

#### Limits of the visual masking paradigm.

When comparing the processing of a clearly visible unmasked stimulus and of a visually degraded masked stimulus, one should be aware of two potential shortcomings. First, the masking procedure induces an inescapable degradation of stimulus processing at a visual stage. Therefore, comparing masked and unmasked stimuli does not amount to a pure comparison between conscious and nonconscious perception, but rather to the comparison between nondegraded conscious information and degraded nonconscious visual information. In other words, one has to keep in mind that any observed difference between the two conditions may include low-level processing differences due to visual degradation itself, upstream from conscious access. Note, however, that in our experimental design, we systematically subtracted activity evoked by the visual masks, in order to isolate the correlates of masked and unmasked words processing.

A second shortcoming of the masking method is that subjects can perform the instructed task only when consciously perceiving the stimuli. Therefore, activation differences between conscious and nonconscious stimuli may relate to the execution of the task downstream from conscious access, rather than to conscious access per se. However, this concern is moderated by evidence that the processing of masked stimuli is sensitive to task instructions and more generally to top-down strategical effects [45–53]. Note also that this issue is not restricted to visual masking, but that it equally applies to most paradigms used for the study of conscious access, including paradigms without visual degradation such as the attentional blink, attentional blindness, or masking at threshold. It is generally not feasible to equate performance with conscious and nonconscious stimuli, probably because improved performance is an integral consequence of the greater availability of information made possible by conscious access. The few paradigms that have attempted to compare conscious and nonconscious processing with equated performance levels have used various means of degrading conscious performance down to nonconscious levels [24].

### Neurophysiological Predictions Derived from the Global Workspace Model

In the light of our model, the masked–unmasked contrast corresponds to a comparison between a visual representation satisfying only condition 1 and a representation satisfying all three conditions for conscious access listed above. The global workspace model therefore leads to the following four predictions.

#### Prediction 1: a common early stage of processing.

Both masked and unmasked words should evoke similar neural activity within an early time window, reflecting a fast feedforward sweep propagating from posterior to anterior cortices. In particular, invisible masked words should induce transient event-related responses along the ventral visual pathway, as assessed by iERPs and ERSP.

#### Prediction 2: a temporal divergence.

Following this initial common stage, only unmasked words should be associated with sustained effects. We thus predict a divergence in cortical activation for unmasked and masked words. Given that we contrasted heavily masked stimuli with unmasked stimuli, we expect a progressive buildup of the divergence between these two conditions. In the light of recent high-resolution scalp electroencephalogram (EEG) studies in visual masking and attentional blink paradigms, this temporal divergence is expected to occur within a 200–500-ms window [1,2].

#### Prediction 3: an anatomical divergence.

The activation of frontal and parietal areas, which are allegedly dense in global workspace neurons, should be particularly sensitive to consciously perceived words (see [32] and Figure 1 of [22] for explicit simulations of this property). Although masked words may cause a small, transient and local activation within these regions, we predict that unmasked words should elicit a global and long-lasting activation of these regions, corresponding to a broadcasting process.

#### Prediction 4: phase synchrony and causality.

During this late time window, the long-lasting and long-distance neuronal assembly specific to conscious processing should be associated with an intense increase in bidirectional interelectrode communication. Measures of phase synchrony and Granger causality should be particularly apt at capturing this phenomenon. The global workspace theory is not yet fully explicit about the exact pattern of neuronal oscillations involved in conscious access. In our initial modeling and theoretical articles, no prediction was stated about which frequencies would be affected by conscious access [22,31]. Increased high-frequency power and long-distance neural synchrony were merely thought to be necessary, but not sufficient, conditions for conscious access, contrasting with the local synchrony induced within modular processors during nonconscious processing [22]. In a recent theoretical review, we further pinpointed an important distinction to be drawn between preconscious and conscious processing, as defined by occipitotemporal loops with local synchrony in the former, and long-distance synchronous loops in the latter [23]—but again without specifying the actual frequency bands involved. Explicit neuronal network simulations of global workspace activity during the attentional blink paradigm demonstrated a link between increased gamma-range oscillations (20–100 Hz) and conscious access [32]. In simulations of inattentional blindness, this rather broad gamma band narrowed down to a predominant band at 40–45 Hz [54]. Those frequency ranges, however, were only determined by the particular choice of simulation parameters for corticocortical and thalamocortical propagation latencies, whose values in humans are not known in detail.

In the light of these considerations, the present study of neuronal oscillations should be considered as an exploration of a broad theoretical proposal, rather than an assessment of a precisely articulated prediction. Actually, theoretical considerations by other groups [55,56], as well as recent experimental measurements [57–62], suggest that for distant cortical areas, synchrony cannot be easily achieved in the high-frequency range, where the oscillation period is short relative to corticocortical transmission delays. Thus, these articles would predict that long-distance synchrony associated with conscious access should preferentially occur in the beta frequency range (13–30 Hz), whereas local recurrence would mostly concern the gamma range (>30 Hz), even for masked stimuli [7].

## Results

### Behavioral Measures of Word Visibility

Unmasked words were consciously reportable, and were categorized better than chance level in a forced-choice categorization task on the emotional valence of words (mean discriminability index *d*′ = 2.24 (+1.14 to +3.04), all individual χ^2^ *p*-values and group analysis Student *t*-test *p*-value < 0.0001). In sharp contrast, masked words were not consciously visible, and forced-choice performance was at chance level for each of the implanted patients (mean *d*′ = 0.02 (−0.18 to +0.27), all *p*-values >0.2). Response times (RTs) were similar across the two masking conditions (*p* > 0.38 in Student *t*-test performed on mean RTs; masked mean RT = 1,640 ms, unmasked mean RT = 1,300 ms).

### Intracranial ERPs

We defined masked effects by subtracting the voltages measured on masked blank trials from those associated with masked word trials. This subtraction allowed us to isolate, on a sample-by-sample basis, activations associated with masked word processing (see Figure 1 and Materials and Methods for our detailed three-step statistical procedure). Unmasked effects were defined similarly by subtraction of the unmasked word and unmasked blank conditions.

Figure 1 shows the anatomical distribution of the 176 reconstructed bipolar montages (“electrodes”) from which we obtained valid data across the ten patients. The bipolar subtraction of nearby recording sites reduced distant influences, including those from the reference electrode, and resulted in a signal tightly localized to the implanted structure. Although measures were obtained for all four lobes, it should be kept in mind that major sectors of dorsolateral and polar prefrontal cortex as well as parietal cortex were not sampled.

Among the 176 electrodes, 24.4% (43 electrodes) showed a significant effect for masked words. These effects were observed across all implanted structures but with a predominance of effects on occipital electrodes: 22/55 (40%) within the occipital lobe, 11/78 (14.1%) within the temporal lobe, 4/24 (16.7%) within the parietal lobe and 6/19 (31.6%) within the frontal lobe (χ^2^ *p*-value = 0.004).

Concerning unmasked words, 68.8% of all electrodes (121 electrodes) showed a significant effect of word presence—a remarkably high percentage, given that electrodes had been placed at clinically relevant sites without consideration of their relevance to our visual stimuli. Unmasked effects were observed across all implanted structures but with a particular emphasis on the frontal lobe: 42/55 (76.4%) within the occipital lobe, 49/78 (62.8%) within the temporal lobe, 12/24 (50%) with the parietal lobe, and 18/19 (94.7%) within the frontal lobe (χ^2^ *p*-value = 0.005). The frontal lobe showed a major difference between trials containing masked and unmasked words: almost all contacts were systematically activated during conscious processing of unmasked words (∼95%), whereas this was not the case during unconscious processing of masked words (∼32%).

In order to better assess the specificity of this last result, we ran an ANOVA to directly compare the impact of masking on the proportion of activated electrodes between occipital and frontal lobes. A main effect of masking was observed (86% versus 36%; *p* < 10^−4^), confirming the larger spatial extension of unmasked activations as compared to masked activations. No main effect was observed between frontal and occipital electrodes (58% versus 63%, *p* > 0.5). Crucially, we observed a significant interaction between frontal and occipital cortices and masking condition (*p* = 0.05), assessing the larger differential activation of frontal lobe between masked and unmasked conditions, as compared to the pattern observed in posterior visual cortex.

Note that this spatial analysis is affected by the nonhomogenous sampling of brain regions, minimizing the contribution of cortical structures that were less frequently implanted. Nevertheless, masked effects were more frequent on posterior than on anterior electrodes, whereas unmasked effects were homogeneously distributed. To demonstrate this point, we examined the distribution of the anterior-posterior (*y*) coordinate, in Talairach space, of the electrodes showing a significant effect, and compared it to the spatial distribution of all 176 recorded electrodes (see Figure S1). For masked words, the spatial distribution of significant electrodes was strongly shifted towards posterior sites (*p* < 10^−6^, Kolmogorov-Smirnov test, relative to the distribution of either the whole set of 176 electrodes or to those showing an unmasked effect). The same analysis conducted on the cumulative distribution of unmasked effects showed a spatial distribution statistically indistinguishable from that of the whole set of electrodes.

**Figure 2:**
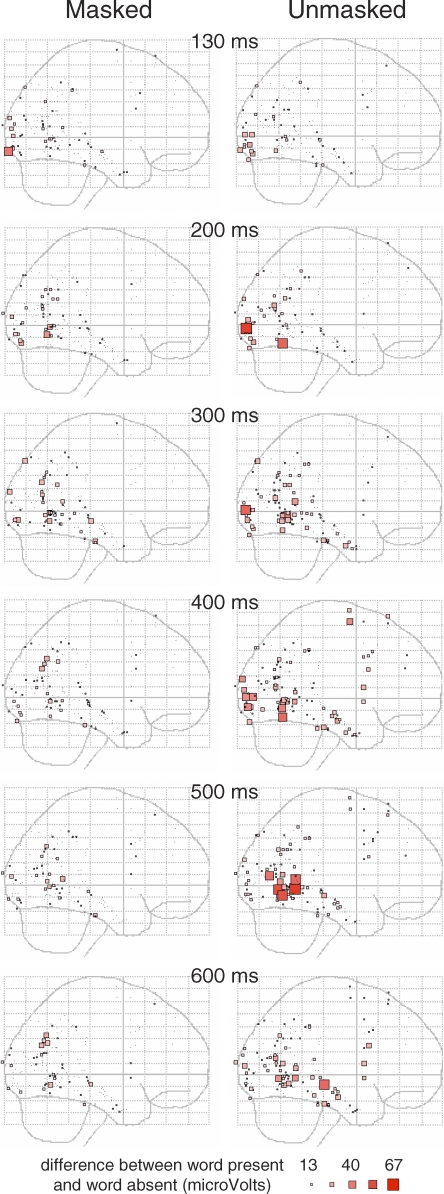
Spatiotemporal Dynamics of iERP Effects Absolute values of iERPs difference (microVolts) between word-present and word-absent in masked (left) and unmasked (right) conditions. Only electrodes showing a significant effect are displayed as red squares. Square size and color intensity are proportional to the absolute voltage difference between the word and blank conditions. Six different time slices are displayed, ranging from 130 to 600 ms (see Videos S1 and S2 for complete videos provided as supplementary on-line material).

Masked and unmasked words were also distinguished by the temporal extension of their activation. A crude analysis, averaging across all electrodes, revealed that masked effects had a mean duration of 60 ms, much shorter than the mean of 378 ms for unmasked effects (*p* < 10^−6^). Masked effects also showed an earlier onset (mean = 366 ms; median = 301 ms) than unmasked effects (mean = 522 ms; median = 497 ms; *t*-test, *p* < 10^−5^). A more relevant analysis focusing on the first significant effect within the subset of electrodes with both masked and unmasked effects showed similar latencies between these two conditions (299 ms and 348 ms, respectively, for masked and unmasked conditions; *t*-test, *p* = 0.30). Indeed, up to approximately 200 ms after word onset, glass brain visualization of the spatiotemporal dynamics of masked and unmasked effects showed a strikingly similar pattern of activations within posterior occipitotemporal cortical regions (see Figure 2). This dynamic pattern is very comparable to the “feedforward sweep” described by Lamme in the nonhuman primate visual cortex using multiunit recordings for latencies up to 100 ms after visual stimulus onset [5]. Clear differences between masked and unmasked effects appeared after 150 ms, with a progressive increase in the intensity and spatial extension of unmasked effects, while masked effects decayed and did not show a similar spatial extension (see Videos S1 and S2).

**Figure 3:**
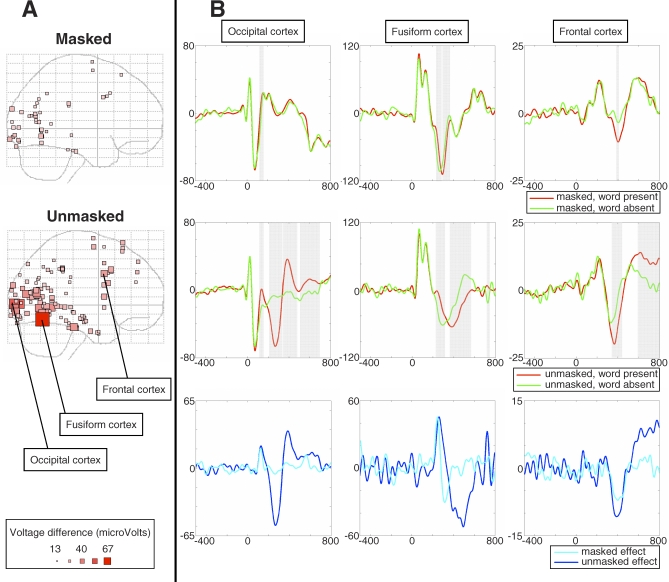
iERP Effects on Three Representative Electrodes (A) Maximum size of significant masked and unmasked effects across the 0–800-ms time window are displayed as red squares, whose size and color intensity are proportional to peak absolute voltage amplitude. (B) Mean iERPs of three representative electrodes in occipital, fusiform, and frontal cortex (location shown in [A]). Shadowed areas indicate significant effects (difference between word and blank conditions). The bottom graphs (blue traces) show the time course of the “word minus blank” subtraction separately from the masked and unmasked conditions. All three sites exhibit an initial common peak, followed by a polarity reversal and delayed activity specific to the unmasked condition.

This general pattern was observed on individual electrodes (see Figure 3). The initial response was often indistinguishable between masked and unmasked effects. This initial common response was usually followed by later effects specifically for the unmasked condition. Out of 14 electrodes showing this pattern with our statistical criteria, 11 of them also showed a polarity inversion of the late sustained effects relative to the polarity of the initial effect.

**Figure 4:**
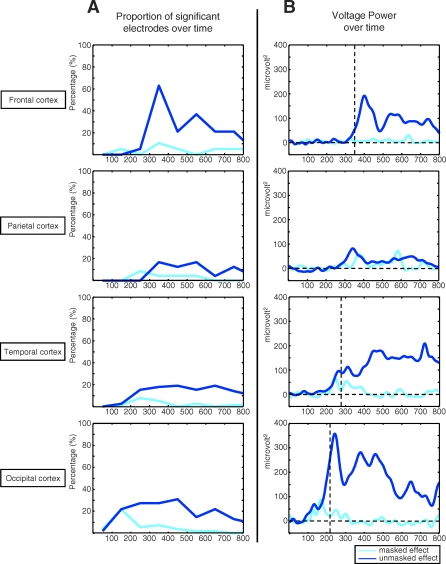
Lobar Analysis of iERPs (A) For each lobe, proportions of electrodes showing a significant effect over time for masked (cyan) and unmasked (blue) conditions, respectively. (B) Voltage power, averaged across electrodes showing at least one significant effect, for masked (cyan) and unmasked (blue) conditions, respectively. Black dashed lines indicate latencies of the first significant differences (*p* < 0.05) between conditions.

A cortical lobe analysis focusing on the proportion of electrodes showing a significant effect over time (Figure 4) showed a similar proportion of electrodes activated by masked and unmasked words at short latencies, whereas at later latencies, the effects were increasingly specific to the unmasked condition. An analysis of the mean voltage power, averaged across electrodes within one lobe, showed a similar temporal dynamics, and additionally allowed us to detect a progressive time delay in the peak of the initial activation common to masked and unmasked words. The time point at which the first significant divergence between masked and unmasked effects occurred, as estimated by a *t*-test (*p* < 0.05), progressively increased from 215 ms to 275 ms and 347 ms, respectively, for the occipital, temporal, and frontal lobes (see Figure 4). The divergence did not reach significance for the small set of 14 parietal lobe electrodes tested (those showing at least one significant effect, masked or unmasked).

### Event-Related Spectral Perturbations

**Figure 5:**
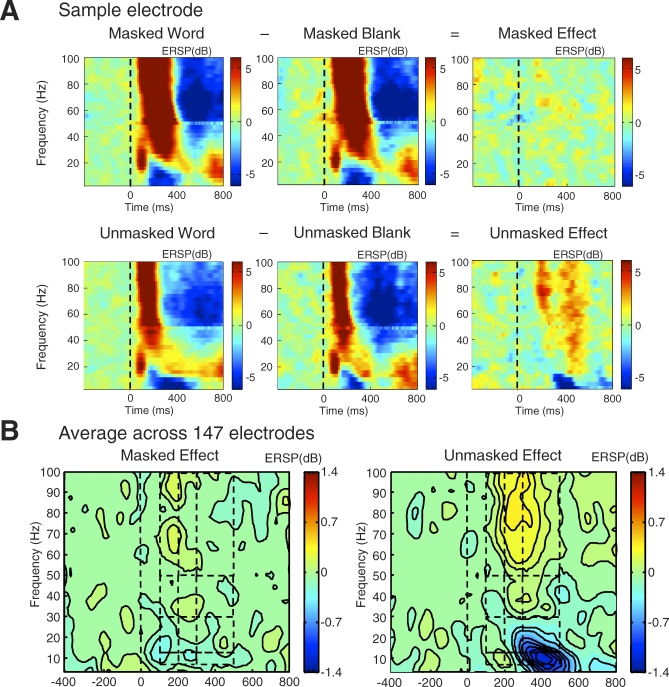
ERSP Effects (A) Time-frequency diagrams showing the ERSPs of a representative electrode (Talairach −19,5, −90, −20). Color indicates the log power increase or decrease in power relative to baseline (same scale in all graphics). Top row, masked words, masked blanks, and their subtraction. Bottom row, same analysis for the unmasked conditions. (B) Time-frequency diagrams of mean ERSPs averaged across 147 electrodes for masked (left) and unmasked (right) effects. Dashed lines delimit the time-frequency windows used for the statistical analyses that appear in Figure 7.

We then turned to frequency-domain analyses of the intracranial signals. Figure 5A shows a typical single-electrode example of the time-frequency transform applied to our data. The masks alone evoked a very strong sequence of event-related increase in the beta and gamma bands accompanied by alpha decrease, followed by a reversal of this pattern. Subtraction of each mask-only condition from the corresponding word-present condition, however, isolated the ERSP induced by the word alone, as a function of whether it was masked or unmasked. As can be seen in this example, masked words induced a slight increase in gamma power 100–200 ms after the stimulus, whereas unmasked words induced a much bigger effect that lasted throughout the epoch and was accompanied by alpha suppression.

**Figure 6:**
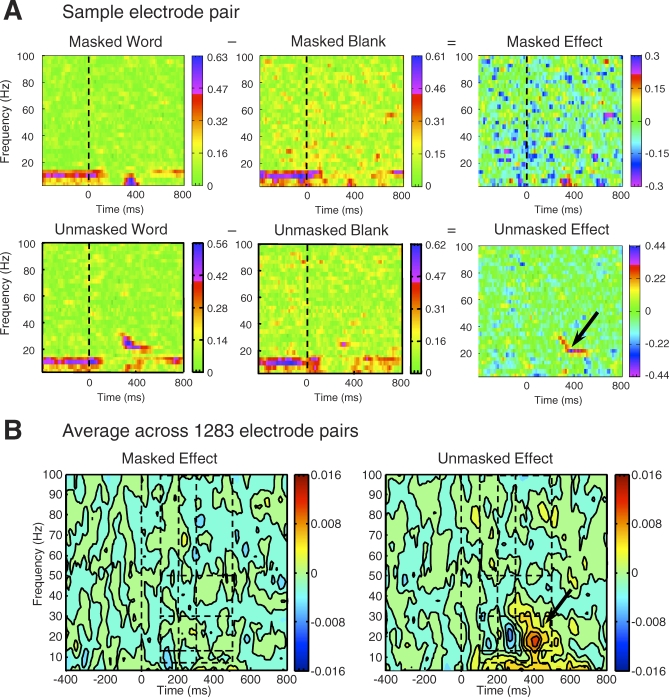
Phase Synchrony Effects (A) Phase synchrony analyses of a representative pair of electrodes (Talairach −12, −97, −12 and −28.5, −77.5, 6). Top row, masked condition; bottom row, unmasked condition. Each picture shows a time-frequency diagram of intertrial phase coherence across the two electrodes (ranging from 0 to 1) for the word condition, the blank condition, and their subtraction (different scale, including negative values). (B) Time-frequency diagrams of ITC averaged across all 1,283 electrode pairs, separately for masked (left) and unmasked (right) effects. Dashed lines delimit the time-frequency windows used for the analyses that appear in Figure 7.

**Figure 7:**
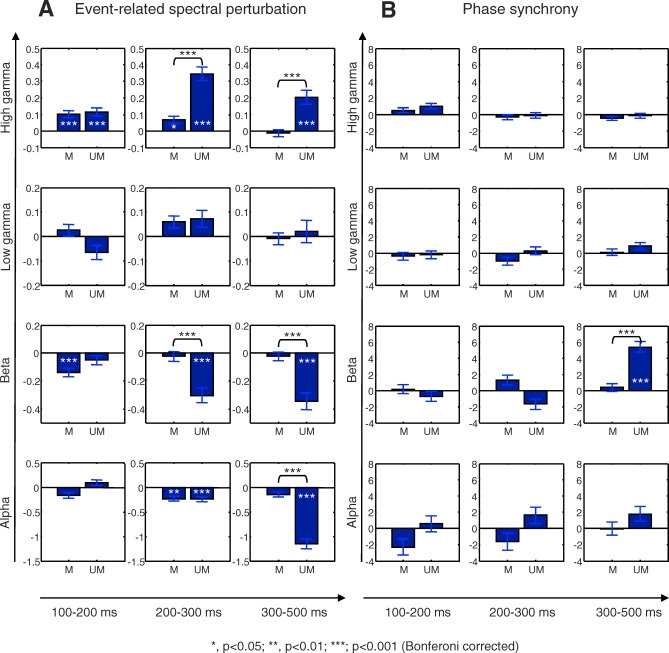
ERSP and Phase Synchrony across Three Time Windows Twelve time-frequency regions of interest were defined on ERSP and phase synchrony–averaged analyses (see Figures 5B and 6B). For each region, mean ERSPs (A) and mean ITC (B) are plotted for three different time windows (abscissa axis: 100–200, 200–300, and 300–500 ms) and for four frequency bands (ordinate axis: alpha = 8–13 Hz; beta = 13–30 Hz; low gamma = 30–50 Hz; and high gamma = 50–100 Hz), separately for the masked (M) and for the unmasked (UM) conditions. Bars represent one standard error of the mean.

To evaluate the generality and significance of such effects, we averaged the time-frequency diagrams across all electrodes (Figure 5B). Statistical comparisons over time-frequency regions of interest, with Bonferroni correction (see Methods and Materials), identified several significant effects. In the 100–200-ms time window, masked words evoked highly significant power changes (beta suppression: *p* = 0.0004; high-gamma increase: *p* = 0.0005). In this time period, there was no significant difference with unmasked words, confirming that a volley of activation, reflected primarily in a gamma increase, can propagate nonconsciously while being largely unaffected by masking [7].

In the next time window (200–300 ms), whereas unmasked words created an even larger power increase in the high-gamma band (*p* < 10^−11^) and decreases in alpha (*p* < 10^−8^) and beta bands (*p* = 10^−5^), masked words induced only small effects of alpha suppression (*p* = 0.0014) and high-gamma increase (*p* = 0.038). Beta and high-gamma bands showed significantly stronger changes for unmasked compared to masked words (all *p* < 0.0007). In the subsequent time window (300–500), alpha suppression, beta suppression, and high-gamma increase were very strong in the unmasked condition (all *p* < 0.0003), but altogether absent in the masked condition, creating a significant difference (all *p* < 0.0002).

In summary, masked words induced significant changes in the power spectrum, particularly increases in the high-gamma band, but these induced oscillations quickly dissipated with time, whereas the ERSPs evoked by unmasked words exhibited a greater power and lasted significantly longer. Note that the above analysis was based on the pooling of ERSP results from all electrodes, regardless of their location. We also replicated this ERSP analysis while separating the electrodes as a function of their lobe of origin. Both the high-gamma increase and late alpha and beta suppression specific to the unmasked condition were replicated within each of the four lobes (see Figure S2). Interestingly, the high-gamma increase peaked earlier in occipital cortex than in temporal, parietal, or frontal, following an approximate posterior to anterior progression (see Videos S3 and S4). Furthermore, the lobar analysis showed that the early nonconscious effects were confined to the occipital and temporal lobes: the only significant effects were a high-gamma power increase in occipital cortex in the 100–200-ms and 200–300-ms windows (respectively, *p* = 0.013 and *p* = 0.016), and decreases in alpha (200–300 ms, *p* = 0.007) and beta (100–200 ms, *p* < 10^−3^) in temporal cortex. In brief, the early ERPS evoked by nonconscious stimuli originated only from occipitotemporal regions, whereas conscious perception was associated with stronger and longer-lasting power changes spreading towards anterior cortical regions.

In this respect, analyses of induced high-gamma power yielded conclusions very similar to those derived from iERP analyses. To better evaluate the relation between induced gamma activity and iERPs, we calculated for each segment for which a significant ERP difference was seen, the absolute value of the iERP effect as well as the mean power evoked in the high-gamma range, averaged over the same time period. A significant positive correlation between these two measures was found, for unmasked words (*r* ^2^ = 14.9%, 241 time segments, *p* < 10^−8^) and, crucially, for masked words (*r* ^2^ = 9.3%, 59 time segments, *p* = 0.028). This means that periods in which a high-gamma–band activity is seen are also periods in which a high-voltage difference between word-present and word-absent conditions exists. In brief, high-gamma activity and iERP are correlated measures that both jointly reflect conscious as well as nonconscious processing stages. Indeed, videos of ERP and of high-gamma activity, provided as supplementary online material, present high similar profiles (see Videos S1, S2, S3, and S4).

### Phase Coherence

Spectral changes are complex phenomena that can be sensitive to local as well as global neuronal synchronization of thalamocortical networks [56]. To evaluate the global workspace model's prediction that access to consciousness is associated with long-distance synchronization, we measured the phase synchrony between all electrode pairs. Phase synchrony can occur independently of changes in induced power: it solely evaluates whether oscillations are reproducibly synchronized across two distant sites in the sense that across trials, they exhibit a systematic phase relationship.

Figure 6 shows a time-frequency diagram of the intertrial phase coherence (ITC) changes induced by the masked and unmasked words, both in an example electrode and in the mean overall electrodes. All statistics were Bonferroni corrected. Statistical analyses revealed no significant coherence changes induced by the masked words. For unmasked words, an increase in beta synchrony in the 300–500-ms time window was highly significant (*t*(1,282 df) = 7.12, *p* < 10^−10^; difference with masked condition, *t*(1,282 df) = 5.43, *p* < 10^−6^). It is particularly interesting to note that (1) this phase synchrony increase was concomitant with a *decrease* in induced spectral power (ERSP) within the same frequency band (see Figure 7A and 7B); (2) no phase synchrony increase was detected in the high-gamma band, although in this band, a highly significant increase in induced power had been detected by ERSP analysis. Thus, ERSP and phase synchrony appear to double dissociate, and beta synchrony appears as a highly selective marker of the late phase of conscious access.

**Figure 8:**
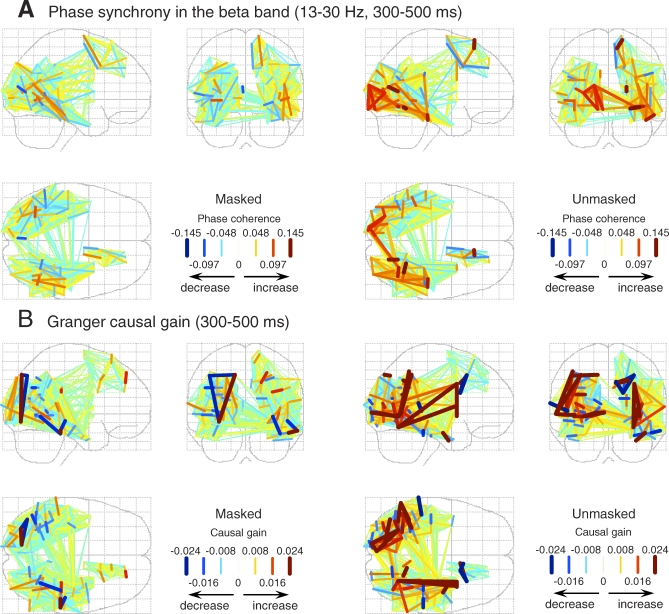
Phase Synchrony and Granger Causal Gain between 300 and 500 ms after Word Onset Each figure depicts three orthogonal views of a transparent “glass brain,” with segments linking, for each patient, all pairs of electrodes. Segments are colored and sized according to the intensity of the increase or decrease in phase coherence in the beta frequency band (A), and in Granger causal gain (B) during the 300–500-ms time window. Superimposed lines are plotted in increasing order of the absolute value of the depicted parameter, so that larger values override smaller ones. Left two columns, masked effects; right two columns, unmasked effects.

Figure 8 shows in graphic form the value of the beta coherence increase due to word presence in the critical time window 300–500 ms, separately for unmasked and masked words. Clearly, unmasked words create a more globally synchronous brain state than masked words. The figure makes apparent that this phase coherence analysis is importantly limited by the available electrodes: we can only analyze coherences between electrodes *within* a given patient, and these tend to be regrouped within a cortical area, thus preventing a thorough analysis of how coherence evolves across distant anatomical sites. For instance, it was not possible to evaluate the prediction that frontal electrodes should cohere more with posterior sites during conscious processing, because in our sample, these two regions were very rarely recorded simultaneously. Still, to probe long-distance connections, we could analyze a subset consisting of electrode pairs in which the two electrodes lie in different hemispheres, thus imposing a long-distance transfer across the corpus callosum. As predicted by global workspace theory, we observed an increase in long-distance interhemispheric beta coherence selective to unmasked words (*t*(71) = 2.50, uncorrected *p* = 0.015). In fact, interhemispheric beta coherence actually decreased when masked words were presented (*t*(71) = 3.14, uncorrected *p* = 0.003), thus creating a strong difference between visible and invisible conditions (*t*(71) = 3.83, *p* = 0.0003).

Conversely, Figure 8 suggests that in the masked condition, there might have been a small local increase in beta coherence within posterior occipitotemporal cortices, which might have been missed in our analysis pooling across all electrode pairs. Indeed, when we restricted only to intrahemispheric electrodes lying within occipital cortex or within temporal cortex posterior to *y* = −20, we detected a significant increase in beta coherence for masked words during the 200–300-ms time window (*t*(734) = 2.34, uncorrected *p* = 0.02), which ceased to be significant in the 300–500-ms time window (*t* = 0.41, not significant [n.s.]). No such increase was seen in other frequency bands, or in other regions (e.g., within frontal electrodes). Thus, nonconscious word processing resulted in only small and barely detectable transient increases in phase coherence within visual cortex, whereas conscious words yielded a massive increase in long-distance beta coherence

### Granger Causality

A final measure of conscious processing that we evaluated is Granger causality [60,63–65], a mathematical tool that can estimate the causal influence that one electrode site exerts on another. Global neuronal workspace theory predicted that access to consciousness for unmasked words would be accompanied by a massive web of causal relations among distant cortical sites, not seen in the masked condition. Granger causality and phase coherence are similar in that both estimate the correlations among pairs of electrodes, but Granger causality looks for temporal contingencies inaccessible to coherence analyses. In a nutshell, the method estimates whether past samples of electrode *j* account for a significant amount of variance in electrode *i*, over and above a simpler “autoregressive” model using only past samples of electrode *i* (see [63] for details). It is possible for two time series to be strongly phase coherent, yet not causally related (for instance, two sine waves with constant phase lag and independent noise). Thus, Granger causality analysis is not redundant with phase coherence analysis: finding that Granger causality increases during conscious perception, perhaps simultaneously with the beta coherence increase, would provide additional evidence in favor of a large-scale reverberating neuronal assembly linking distant sites. Furthermore, unlike phase coherence, Granger causality is a directional measure: it is possible for electrode *j* to causally influence *i* without *i* causally influencing *j* (although it is also possible for two signals to exert mutual causal influences on each other). This analysis therefore provided an opportunity to examine the top-down versus bottom-up propagation of activation during conscious and nonconscious processing.

**Figure 9:**
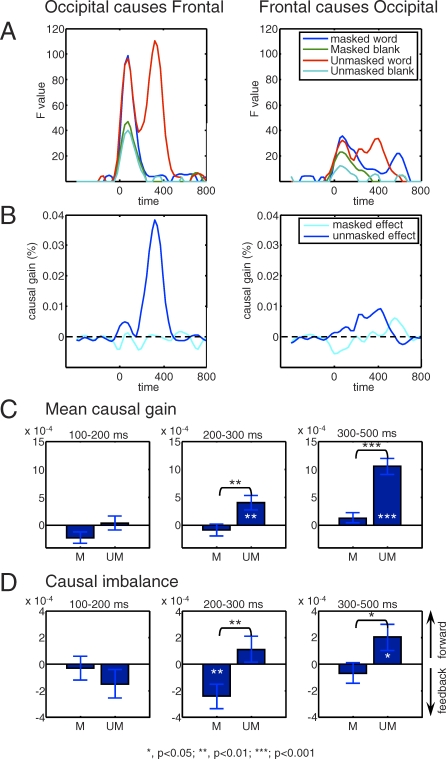
Granger Causality Analysis (A and B) Illustration of Granger causality analysis for a representative pair of electrodes located respectively in the frontal and occipital lobes. For each of the four experimental conditions, an *F*-test evaluates, over a sliding timing window, the causal influence of occipital activity on frontal electrode activity and vice versa (A). Note that this F-test is not directly comparable across conditions (because of smaller number of trials in the blank control conditions), nor can it be taken directly as a test of significance (because of inflation due to auto-correlation [63]). Furthermore, masks alone obviously induce increases in causality. To evaluate how words and their conscious perception affect Granger causality, causal gain was then computed as the difference in the percentage of word-absent (blank) condition (B). Here, an obvious imbalance is seen, with a massive increase in causality only in the occipital-to-frontal direction and in the unmasked condition. For statistical analysis, we distinguished the mean causal gain (averaged across the two directions of causality) and the causal imbalance (difference in causal gain over the two directions of causality). (C and D) show the mean results, averaged over all electrode pairs (bars indicate one standard error of the mean). Mean causal gain and mean causal imbalance were calculated separately across three time windows (100–200 ms, 200–300 ms, and 300–500 ms) are plotted separately for the masked (M) and for the unmasked (UM) conditions.

As a concrete example, Figure 9 illustrates the causality analysis of a sample electrode pair consisting of one frontal and one occipital electrode. At the time of stimulus presentation, a massive increase in Granger causality is seen in the feedforward, occipitofrontal direction (Figure 9A, left panel) and, to a smaller extent in the top-down, fronto-occipital direction (Figure 9A, right panel). Importantly, the curves showing the evolution of the *F*-test for causality as a function of time exhibit two successive peaks: one early peak is evoked by the masks alone (146 ms after mask onset), whereas a second peak (325 ms after word onset) is seen only when a word is present and unmasked. As illustrated in Figure 9B, a strong “causal gain” is therefore observed, approximately 200–450 ms after word onset, when the word-present condition is contrasted to the word-absent condition. This effect is seen mostly in the feedforward direction, thus engendering a “causal imbalance” (higher causal gain in one direction than in the other).

Similar increases in causal gain and causal imbalance in the unmasked condition were seen in a large set of electrode pairs. To evaluate their statistical significance, we first averaged the causal gains across all electrode pairs and both causal directions, separately for masked and unmasked conditions, and used *t*-tests to evaluate the significance of changes within three temporal windows (100–200, 200–300, and 300–500 ms, similar to the ERSP and phase coherence analyses, with Bonferroni correction over the number of windows tested) (see Figure 9C). A massive increase in mean causal gain was observed during the 300–500-ms window in the unmasked condition (*t*(1805) = 7.60, *p* < 10^−13^), but not in the masked condition (*t* = 1.47, n.s.), resulting in a significantly larger causal gain during conscious than during nonconscious processing (*t*(1805) = 5.46, *p* < 10^−8^). The effect was already perceptible in the 200–300-ms window, though it was much smaller (unmasked: t(1805) = 3.03, *p* = 0.0075; masked, *t* = −0.86, n.s.; difference: *t*(1805) = 2.89, *p* = 0.012). No effect reached significance in the 100–200-ms window.

Figure 8B illustrates the anatomical distribution of the mean causal gains during the 300–500-ms window. In the unmasked condition, causal relations increased massively among many distant sites, both within the occipitotemporal cortex, between occipitotemporal cortex and distant frontal and insular sites, and across the corpus callosum. By contrast, increases were very scarce in the masked condition and never reached significance even when restricted to posterior electrodes only.

With similar methods, we evaluated the statistical significance of changes in the variable of “causal imbalance,” which is the subtraction of forward causal gain minus backward causal gain in the same electrode pair. This variable evaluated the dominant directionality of causality (posterior to anterior = feedforward, or anterior to posterior = feedback). During the 300–500-ms time window, in the unmasked condition, there was a small imbalance with a higher causality gain in the feedforward compared to the feedback direction (*t*(1,850) = 2.07, *p* = 0.039 before Bonferroni correction). Although marginally significant, this finding occurred in the predicted late time window and fits with our prior hypothesis that during this time period, and only in the unmasked condition, perceptual information gains access to consciousness and is therefore able to invade anterior areas in a feedforward manner. Indeed, in this time window, the imbalance was not significant for masked targets (*t* = −0.90, n.s.), creating a difference for unmasked as opposed to masked targets (*t*(1,805) = 2.09, *p* = 0.037 before Bonferroni correction).

Quite surprisingly, however, in the preceding time window (200–300 ms, see Figures 9D and S3), there was a significant imbalance in the converse direction (higher causality in the top-down or feedback). This was true only for the masked condition (*t*(1,805) = −2.66, *p* = 0.024, Bonferroni corrected), not the unmasked condition (*t* = 1.11, n.s.), a significant difference (*t*(1,805) = 2.70, *p* = 0.021 Bonferroni corrected). This unexpected finding, further discussed below, may indicate that in the masked condition, there is a top-down component of attentional amplification, perhaps relating to an unsuccessful effort to identify the masked word.

## Discussion

We first summarize and discuss our results in the light of the four theoretical predictions listed in the introduction. We then focus on the nonpredicted results and discuss two important limitations of this study. Finally, we propose a description of the neural signature of conscious access combining the four neural measures that we could gather here within the same subjects.

### Discussion of the Four Predictions

#### An early stage of processing, common to masked and unmasked stimuli.

Masked iERP effects shared common temporal properties: most of them (75%) exhibited a short latency and a transient nature. As indicated in Figures 2, 3, and 4, masked and unmasked iERPs were strikingly similar at short latencies (see glass brain at 130 ms on Figure 2, and initial parts of the curves in Figure 4). Masked words also induced early (100–200 ms) spectral power effects corresponding to significant beta suppression and high-gamma band increases. Again, in this early time period, no significant difference was found between masked and unmasked ERSPs. iERP correlates of the processing of masked words were observed in almost 25% of the 176 electrodes, across all cortical lobes. These effects were not exclusively confined to posterior areas, but reached the most-anterior implanted structures, including prefrontal cortex.

Taken together, these results are highly suggestive of a feedforward mode of processing that propagates nonconsciously and is initially largely unaffected by masking. This experimental validation of our prediction 1 also corresponds to an extension to the human brain of the concept of “feedforward sweep” proposed by Lamme and Roelfsema for the macaque brain [11].

#### A temporal divergence.

We observed a major divergence, as predicted, between the processing of masked and unmasked words. This divergence occurred around 200 ms for the most-posterior implanted electrodes. Although both masked and unmasked words evoked similar initial cortical activity, only consciously visible words evoked long-lasting effects. iERPs evoked by masked words decayed rapidly around 200 ms for occipital electrodes and around 300 ms for frontal electrodes. Accordingly, clear differences in spectral power were also observed. Initially (100–200 ms), increases in spectral power in the high-gamma band were jointly elicited by masked and unmasked words, but in the 200–300-ms window, a clear difference occurred and only unmasked words still induced significant effects within the late temporal window (300–500 ms).

Recordings of single-unit activity in other epileptic patients indicate that single neurons within the human temporal lobe encode an invariant representation of stimulus identity [66]. A recent study using a backward-masking paradigm has shown that these neurons are strongly activated during conscious perception [67]. The onset latencies of these neurons are mostly around 400 ms [68], supporting our present observation that neural representations of conscious percepts are activated during the late phase (300–500 ms) of stimulus processing.

The observation of a common level of activation followed by a clear divergence between masked and unmasked conditions bears upon the debated issue of qualitative versus quantitative models of conscious perception (for discussions of the quantitative activation strength theory, see [69–72]). Indeed, a fine temporal description of the brain activity associated with masked and unmasked stimuli is essential to resolve this theoretical issue. If we just compared the net activation summed across time, as might be reflected in the fMRI BOLD signal, we would find a difference of intensity between the two conditions, compatible with both qualitative and quantitative models (e.g., see occipital electrodes in Figure 4B). However, the fine temporal description reported here refutes purely quantitative models of conscious perception, by showing an initial common intensity followed by a clear divergence, in line with the predictions of nonlinear models of conscious access [1,2,32,54].

Although we noted above that our observations fit with global workspace theory, they are also largely in line with the feedforward/feedback model advocated by Lamme and Roelfsema [11]. According to this model, an initial feedforward sweep does not differentiate nonconscious and conscious processing, but recurrent feedback loops uniquely characterize conscious processing. Yet a major difference between our results and those of Lamme and Roelfsema concerns the timing of feedback processing. Lamme and Roelfsema observed an early divergence in V1, around 100 ms in nonhuman primates [4,26] and 120 ms in humans [9], and they suggested that virtually any recurrent loop, even locally within visual cortex, might suffice to cause conscious-level processing [5]. Zeki [3] and Pins and Ffytche [6] also emphasize early visual events as the main determinants of conscious visual experience. On the other hand, we essentially observed late differences between masked and unmasked effects, and our theory proposes that it is only the late and global patterns of recurrent activity, particularly those involving prefrontal cortex, that correspond to conscious-level processing. None of our four analyses (iERP, ERSP, synchrony, and causality) ever detected any event specifically associated with conscious reportability before 150 ms, and in most of them, the main differences were found after 300 ms.

One possible explanation for our observation of a late divergence may originate in the complexity of the stimuli and in the areas being recorded. Visual word comprehension necessitates at least 250 ms in humans, and occurs in areas well beyond V1 in the hierarchy of cortical visual pathways. With simpler stimuli (e.g., line gratings or Gabor patches), the divergence may occur earlier. Yet, we consider this possibility unlikely given that our main contrast simply asked about the conscious or nonconscious coding of word presence (by contrasting word versus blank trials), a distinction that could have been seen as early as in V1.

It could also be argued that we did observe a difference in masked versus unmasked iERPs at some occipital sites around 150 ms (Figure 4), and that only this early divergence is critical for conscious perception, whereas all the other later measures only reflect differences in subsequent word processing. Yet this possibility is made unlikely by the results of other paradigms such as the attentional blink [73] or inattentional blindness [74]. During attentional blink, when the very same stimuli are classified subjectively as being consciously or nonconsciously perceived, only a late divergence is observed by EEG [2] or by magnetoencephalography (MEG) [61]. Early visual events such as the P1 and N1 are strictly undiagnostic of conscious reports [2].

#### Anatomical divergence and involvement of prefrontal cortex.

In close accordance with our broadcasting prediction, frontal lobe electrodes were characterized by a different pattern for consciously and nonconsciously perceived words. During conscious processing of unmasked words, iERPs showed a significant deviation on almost all electrodes (18/19), whereas the voltage differences of these same electrodes remained remarkably low for masked words (see Figure 4). Electrodes on other lobes did not show such a dichotomy (from 50% to 76% of active sites with unmasked words). A similar anatomical divergence was seen with ERSPs: in the masked condition, high-gamma increases were only seen in posterior occipital and temporal electrodes, whereas they occurred in all four lobes in the unmasked condition.

Those observations fit with the global workspace model, which postulates that once a representation is consciously accessed, a broad distributed network, involving in particular prefrontal cortex, ignites and broadcasts its content. Computer simulations of this ignition process within thalamocortical networks have shown strong recurrent interactions occurring exclusively during conscious access, implying that the relevant neurons are suddenly coactivated in a cooperative manner and emit increased and synchronized high-frequency oscillations in the gamma range [32,54].

An important qualification, however, is that the 19 frontal electrodes were recorded in only two patients, and that the present study did not sample homogeneously from frontal and parietal structures. Indeed, frontal electrodes were almost confined to mesiofrontal and peri-insular regions, whereas dorsolateral prefrontal cortex was simply not implanted in our sample. In the future, obtaining a better sampling will be important in order to determine whether the ignition property is presented in specific subareas of prefrontal cortex and in which order they activate.

Beyond the sudden and massive involvement of prefrontal cortex in conscious access, the hypothesis that conscious information is “broadcasted” widely in the cortex is also supported by the incidental observation that although electrode location was only determined by clinical considerations, up to 68% of electrodes showed an unmasked effect. This proportion is remarkably high and indicates that a piece of visual information (here, the presence or absence of a written word), although initially coded only locally within occipital cortex, can eventually influence a great variety of distant cortical areas. This distributed influence was exerted only in the conscious case, since only 24% of electrodes were affected by masked words.

Naturally, the observation of a voltage deviation to a visible word does not imply the existence, within each of the sampled sites, of neurons specifically tuned to processing of that word. Rather, the global workspace model postulates that activation coding for conscious-level information is distributed broadly by pyramidal neurons with long and branching axons arising from layer II/III neurons, which are particularly dense in prefrontal and cingulate cortex [75]. Diffusion of workspace activation along those axons would lead to a measurable depolarization in the dendritic trees of many target neurons, as observed here, even if many of those target neurons need not fire in response to this “broadcast.” According to Shanahan and Baars [76], the broadcasting property of the workspace architecture, by allowing many areas to simultaneously receive the same signal and, in parallel, determine its relevance to them, may provide the first elements of a solution to the “frame problem,” i.e., the fast shifting of information relevant to the organism's goals. Whatever its ultimate functional interpretation, the present observation shows clearly that conscious information is indeed made available simultaneously to many cortical sites. In the future, replications studies in epileptic patients implanted with both iEEG electrodes and with microelectrodes appropriate for single-unit recording could, in principle, dissociate action potentials from postsynaptic dendritic integration, as was observed in macaques [77,78]. Recent single-unit recordings in epileptic patients support the feasibility of such developments [66–68,79].

#### Phase synchrony and causality.

In our data, only the unmasked stimuli were associated with a significant increase of phase synchrony between distant pairs of electrodes in the beta range of stereoelectroencephalographic (SEEG) frequencies (13–30 Hz, peaking around 20 Hz). This effect occurred in the late 300–500-ms time window and was simply absent for masked words. This result supports our prediction that conscious access involves an improved long-distance exchange of information across a very broad cortical network. Interestingly, this increase of coherence between distant brain regions occurred during the same temporal window where long-lasting iERP effects and late ERSP effects were seen (alpha and beta suppression and high-gamma increase). This finding suggests that all three electrophysiological measures may reflect different aspects of a single phenomenon. Long-distance coherence between distant regions appears to co-occur with the active maintenance of locally coded representations.

The last measure that we used to assess long-distance communication between distant cortical sites examined the causal relations between pairs of electrodes. We observed an increased causal gain exclusively for the conscious processing of unmasked stimuli during the 200–500-ms time window. Again, late causal changes (>300 ms) were simply absent for masked words. The conscious increase in causal gain was bidirectional, but with a small and marginally significant causal imbalance suggesting that the dominant direction of causality was from posterior to anterior electrodes. This observation is consistent with the global workspace theory's prediction that conscious access occurs during a late time period and is dominated by an inflow of perceptual information from posterior visual areas into hierarchy higher frontal and parietal cortices [22,23,31,32].

Given that all recordings were obtained from patients with focal epilepsy, it could be argued that Granger causality results were affected by the epileptic focus driving surrounding brain regions. However, several considerations minimize the impact of this potential physiological artifact. First, electrodes providing abnormally ample voltage in more than 5% of trials, corresponding mostly to epileptic events, were systematically excluded from our analyses. Second, all trials were submitted to stringent rejection criterion, based both on voltage amplitude and on spectral time-frequency plots. Third, observing a strong occipital to frontal causal imbalance across all electrodes gathered from all patients suggests that this pattern is independent from the specific localization of their various epileptic foci. Fourth, this hypothesis would require a replicable time-locking of epileptic activity with respect to the onset of visual stimulation across patients, an infrequent and unlikely possibility. Still, it will be important to replicate the causality analysis in normal controls, using either MEG or high-density EEG measures.

### Nonpredicted Results

#### Beta-band rather than gamma-band phase synchrony.

As noted in the introduction, neuronal simulations of the global workspace architecture predicted that conscious access would correlate with increases in phase synchrony at frequencies within a large gamma band ranging from 20–100 Hz, centered around 40 Hz [32,54]. Actually, such increases in synchrony appeared in the beta frequency range (13–30 Hz), where spectral power decreased, rather than in the gamma range where the largest increases in induced power (ERSP) were observed.

The present finding, however, is not an isolated one. In the macaque monkey, Buschman and Miller, in their study of top-down versus bottom-up shifts in attention [62], found a greater increase in a low-frequency band (22–34 Hz) for top-down shifts of attention and a greater increase of synchrony in a higher band (35–55 Hz) for bottom-up shifts. Most directly relevant to conscious access, Gross et al. [61], using MEG in humans, observed that the main correlate of target visibility during the attentional blink paradigm was a massive change in beta-band synchrony across distant frontal and parietal sites. Intracranial recordings in macaques and epileptic humans showed a similar pattern, with local gamma-range synchrony and long-distance beta-range synchrony [57–59]. Note also that several studies dedicated to the binding problem in perception [80] seem compatible with this explanatory framework. The first experimental contribution to the hypothesis of assembly coding signaled by gamma oscillatory synchrony came from recordings in area 17 of the anesthetized cat [81]. Recordings in distant areas in the cat, and scalp EEG studies in humans showed long-range coherence in low-frequency bands (theta and alpha, 4–12 Hz), whereas local coherence effects were predominantly observed within the gamma band [82,83]. Likewise, von Stein and colleagues suggested, on the basis of local field potential (LFP) data in cats, that long-distance top-down effects could be mediated by middle band activities (4–30 Hz), whereas local bottom-up effects would affect gamma-band frequencies [82].

At the theoretical level, a systematic relation may indeed exist between the size of a neuronal assembly and the frequency at which it can sustain synchronized oscillations. As suggested by Pascal Fries, gamma oscillations in cortical areas, with periods of 10 to 30 ms, may synchronize only in the face of short conduction delays (1–3 ms) and may therefore contribute to locally coded thalamocortical representations [55]. When the recorded neuronal groups are more distant, phase-coherent oscillations are often found in the lower-frequency beta range [57–62], probably because their slower period (30–80 ms) allows them to resist the longer conduction delays (5–15 ms) needed to bridge large cortical distances. Thus, beta-coherent oscillations may preferentially subserve long-distance synchronization and broadcasting. In the future, it will be important to incorporate this effect in the global workspace simulations, as well as to account for the observed decrease in power that accompanies the increase in beta synchrony in our data.

#### Late effects during nonconscious processing.

Most (75%) of the iERP effects elicited by masked words were confined to an early time window (<500 ms), in accordance with the usual fleeting lifetime of unconscious processes. However, a rather high proportion (25%) of these unconscious iERP effects were still observable and significant at latencies around 600–700 ms after word onset. This result replicates and extends our previous observation of late unconscious modulations of amygdala activity by the emotional valence of masked words in a close but distinct paradigm [43]. These late unconscious effects call for theoretical refinements, as the global workspace model and many other proposals predicted a fast decay of subliminal activity [22,33,84]. We may conceive of at least two nonexclusive explanations. First, these late effects may reflect a reverberation of activation within local cortical or thalamic loops, enabling their maintenance over an unusually long time window. Note, however, that neither phase synchrony nor Granger causality measures could detect any local reverberations for masked stimuli beyond 300 ms (see Results), raising the issue of their level of sensitivity. Second, subjects were actively engaged in the forced-choice discrimination task and tried to overcome visual masking by focusing their attention on the stimuli, as reflected in their long RTs (see Results). Such top-down attentional amplification of perceptual processors may improve their ability to maintain the fleeting stimulus information and thus cause a late iERP effect for masked words. Indeed, the existence of such top-down effects on subliminal processing was inferred from behavioral measures in other masking paradigms [45].

#### Reverse pattern of causality during nonconscious processing.

Interestingly, a reversed pattern of causality was observed for masked words during the intermediate-latency time window (200–300 ms), with causal relations now flowing from anterior towards posterior electrodes. This observation was unpredicted. However, it may again be explained by an effortful top-down attentional amplification of the masked stimuli, thus creating a temporary flow of activity between anterior regions and posterior visual processors. Several studies have previously noted how attention and consciousness differ, in that attention can be oriented towards nonconscious stimuli and influence them without yet allowing them to cross the threshold for conscious access [23,45,46,53,85,86]. In this respect, our results bear similar with those of Buschman and Miller [62], who used single-cell recordings in the awake monkey to demonstrate a top-down frontoparietal sequence of activity accompanied by beta frequency increases during a difficult visual search task (although their study did not evaluate consciousness or Granger causality).

Note that the calculation of causality requires computing a temporally shifted regression over a relatively large time window (here, 320 ms). Thus, it is likely that only temporally sustained causal effects can be detected with this index. This feature may explain why the initial nonconscious feedforward sweep that we inferred from iERPs did not lead to a measurable causal gain with a posterior to anterior causal imbalance. Our causal gain measure seems sensitive only to long-lasting causal effects, and largely blind to fast and transient propagations of neural activity.

### Limits of the Present Paradigm

It is important to keep in mind the limits of the present paradigm. First, when comparing the neural activity elicited respectively by a masked word and by an unmasked word, as was done here, one is actually comparing the correlates of a conscious representation with those of a degraded nonconscious representation. Early differences are therefore not guaranteed to isolate a neural signature of conscious processing per se [23]. Indeed, Del Cul et al. [1] demonstrated that early visual components such as the P1b, N1, or N2 are affected by masking, yet do not meet the criteria for a strong one-to-one correlation with subjective reports of conscious experience. It therefore seems likely that the early iERP differences that we observed at occipital sites only reflected the early and not-yet conscious effects of masking (see, for instance, the peak of activity observed on occipital lobe electrodes on Figure 4, bottom right panel). The three additional markers of conscious processing that occurred later on, around 300 ms, synchronous to the activation of frontal regions, appear to be more consistent with the existing literature on electrophysiological correlates of conscious access [1,2,61]. Indeed, they concur nicely with ERP results obtained in the attentional blink [2,61], a paradigm that may be more appropriate for answering the early versus late debate because it involves an undegraded stimulus that is only made invisible by central competition.

A second limitation relates to task confounds. In the present work, some of the observed differences might correspond to differences between a task being performed (in the conscious condition) and a task not being performed (in the nonconscious condition). Once a word was consciously perceived, subjects were able to complete the requested semantic emotional categorization task, a complex decision that presumably requires high-level coordination of multiple brain areas. In the masked condition, on the other hand, although subjects still responded to the task, objective performance was by chance. Some of the neurophysiological differences that we reported between masked and unmasked words might therefore be related to task performance rather than to conscious access itself.

Mitigating this criticism, however, is the fact that our task appeared to be more difficult for masked words than for unmasked ones, as indicated by longer RTs and by informal subject reports. Indeed, the observed imbalance in the top-down direction for masked words not observed for unmasked words in the 200–300-ms window suggests that nonconscious processing was not free of attentional effects.

Nevertheless, an important direction for future research will be to minimize task differences while still comparing neural activity associated with conscious and nonconscious perception. Passive viewing of stimuli could be a valuable instruction, but does not guarantee an absence of task differences (indeed, it seems obvious that subjects would continue to read and to memorize the conscious words, but not the nonconscious words). The task problem is notoriously difficult because performance levels are very rarely identical under nonconscious and conscious conditions. Nevertheless, using “blindsight” conditions with unseen but better than chance performance, matched with seen but degraded performance, Lau and Passingham [24] used fMRI to identify an activation of dorsolateral prefrontal cortex that was specifically associated with conscious experience. This type of paradigm may be usefully complemented with intracranial recordings.

We close this issue with a general consideration, initially put forward by phenomenological philosophers [87]: whenever a subject is conscious, he is necessarily conscious of a given mental content. Consciousness is an transitive or “intentional” process (it is “about” a certain content), and therefore it may be illusory to look for a “pure” form of consciousness independent of its particular contents and of the tasks that it affords. Applied to neuroscientific experiments, this property of consciousness implies that when imaging a brain having some conscious experience, we will necessarily observe activations corresponding to a specific conscious content. Nevertheless, satisfactory solutions to overcome this major limitation may exist. For instance, it could be particularly relevant to replicate the present results while manipulating the subjects' task. This track of research may look for the common and invariant correlates of conscious access, irrespective of the task being performed and of the specific mental content being probed. Plausible candidates include late brain-scale activation and beta synchrony, since they were observed in the present results as well as other EEG and MEG studies of conscious perception [1,2,61], with various tasks of letter perception, word reading, or digit comparison.

### Toward a Neural Signature of Conscious Access

The main motivation of our study was to probe the convergence of multiple neurophysiological measures of brain activity in order to define candidate neural signatures of conscious access. Conscious word processing was associated with the following four markers: (1) sustained iERPs within a late time window (>300 ms after stimulus presentation); (2) sustained and late spectral power changes, combining a high-gamma increase, beta suppression, and alpha blockage; (3) sustained and late increases in long-range phase coherence in the beta range; and (4) sustained and late increases in long-range causal relations.

Our results suggest that in the search for neural correlates of consciousness, time-domain, frequency-domain, and causality-based electrophysiological measures should not be seen as competing possibilities. Rather, all of these measures may provide distinct glimpses into the same distributed state of long-distance reverberation. Indeed, it seems to be the convergence of these measures in a late time window, rather than the mere presence of any single one of them, that best characterizes conscious trials. For instance, masked words also elicited significant iERPs and significant increases in spectral power in the gamma band, contemporary with short-range synchronies in the beta range during the 200–300-ms window. Yet, those words were not consciously accessed, which implies that neither iERPs (even those recorded from frontal cortex), nor gamma-band activity or beta synchrony per se are unique markers of conscious experience. Our results suggest that only late sustained long-distance synchrony and late amplification (>300 ms) may be causally related to conscious-level processing.

There are yet other mathematical measures derived from nonlinear dynamics that could have been applied to our dataset, such as dimensional activation [88] or neural complexity [21,89], although some of them remain to be made operational in a computationally tractable manner. We consider it likely that these measures would also show a pattern unique to conscious perception. The present work suggests that, rather than hoping for a putative unique marker (*the* neural correlate of consciousness), a more mature view of conscious processing should consider that it relates to a distributed pattern of brain activation that occurs at a specific level within a complex anatomical and functional architecture, and that it can therefore be reflected by many partially overlapping physiological measures.

## Materials and Methods

### Ethics statement.

Experiments were approved by the Ethical Committee for Biomedical Research of Pitié-Salpêtrière Hospital in Paris (agreement #99–04 issued on 15 December 2004), participants gave informed consent, and all clinical investigation have been conducted according to the principles expressed in the Declaration of Helsinki.

### Patients.

Ten patients (five men) suffering from drug-refractory epilepsy were stereotactically implanted with depth electrodes as part of a presurgical evaluation. One patient was implanted twice. Patient ages ranged from 18 to 47 y. Eight patients were right-handed; one man was left-handed; one woman was ambidextrous. Neuropsychological assessment revealed normal or mild impairment in general cognitive functioning: verbal IQ ranged from 65 to 97 and performance IQ from 64 to 120.

### Experimental protocol.

On each trial, patients were randomly presented either with a masked word (33%), a masked blank (17%), a visible word (33%), or a visible blank (17%). A total number of 548 trials were presented for each patient. Words or blanks were presented for 29 ms and were preceded by a visual forward mask (string of hash signs [#]) presented for 71 ms, and were followed by a backward mask (string of ampersands [&]) presented for 400 ms (see Figure 1). Words or blanks were made visible by simply removing the backward mask. Although mask removal in itself affected brain activity, in order to discard the activation induced by the masks, we systematically subtracted each word-present condition with its corresponding blank condition, thus isolating the processing path of the masked or unmasked word.

In order to maximize attentional engagement and word processing, participants were engaged in a forced-choice task of categorizing each word as threatening or nonthreatening, even on the masked trials. Subjects responded by manually pressing one of two response buttons with the left and right index fingers, and hand response instructions were inverted halfway through the experiment. To prevent automatic stimulus–response learning, we used two distinct sets of 92 French words each for the masked trials and the visible trials, so that the masked words were never seen consciously. In each list, half of the words were threatening (e.g., *danger*, *kill*), with variable frequencies, lengths (three to eight letters) and lexical categories (verbs and nouns). The other half included nonthreatening, emotionally neutral words (e.g., *cousin*, *see*), matched for frequency, length, and category.

### Behavioral measures.

RTs of less than 250 ms or more than 5,000 ms were discarded. Median RTs were calculated for each patient and for each condition. RTs were compared across conditions using analysis of variance (ANOVA) *F*-tests. For each patient, we also computed objective discriminability (*d*′) separately for each of the two masking conditions. We then assessed better-than-chance performance on *d*′ using both individual and group statistical criteria. First, individual χ^2^ statistics were calculated for each condition: proportions of correct and incorrect responses to emotional and neutral words were compared with those in the expected random distribution. Second, group analysis was performed using a *Z*-test testing distributions of *d*′ against a zero-centered Gaussian.

### Intracranial recordings.

Patients were implanted intracerebrally with depth electrodes, each bearing four to eight recording sites (Ad-Tech Medical Instruments). Recording sites were 2.3-mm long, 1-mm diameter cylinders, separated by a distance of 10 mm. The structures to be explored were defined on the basis of ictal manifestations, electroencephalography (EEG), and neuroimaging studies. For each recording site, the Cartesian coordinates (*x*, *y*, *z*) were calculated after normalization of the anatomical three-dimensional spoiled gradient recalled (SPGR) anatomical cerebral MRI into Talairach space using SPM2 (Matlab).

### iERPs processing.

iERPs were digitized at 400 Hz, referenced to the vertex (Nicolet-BMSI). Epochs were then extracted (−500 ms plus 1,000 ms from word onset), submitted to automatic artifact rejection (±300-mV threshold), visually inspected, and notch filtered (50 Hz) using EEGLAB software (Matlab) [90]. Recording sites with more than 5% of rejected trials were discarded from analysis. To maximally prevent the measurement of the same electrical signals by multiple sites, for instance, through the common vertex reference, bipolar montages were calculated by subtracting the signals recorded from adjacent sites belonging to the same-depth electrode. This calculation resulted in a total of 176 bipolar recordings (here called “electrodes” for simplicity) across ten patients (corresponding to 11 distinct implantations). For each bipolar montage, the Cartesian coordinates (*x*, *y*, *z*) were calculated as the medium location of the two adjacent recording sites, resulting in the following anatomical repartition: 55 electrodes in the occipital lobe, 78 in the temporal lobe, 24 in the parietal lobe, and 19 within frontal lobes. For simplicity, in the remnant of this paper, we refer to these recomputed bipolar montages as “electrodes.” Eighty-two electrodes were recorded within the left hemisphere and 94 in the right hemisphere. Baseline correction (from −500 to 0 ms before word onset) was applied, and potentials were averaged from −500 up to 800 ms in order to keep the same temporal windows for ERPs and time-frequency measures.

We used a three-step strategy to assess the statistical significance of our results. First, the ERPs of masked words or of unmasked words were compared with those of masked or unmasked blanks by using sample-by-sample *t*-tests, with a criterion of significance being set at *p* < 0.001 for a minimum of five consecutive samples. Second, we further checked the statistical significance of the observed effects (number of consecutive samples with *p* < 0.001 on *t*-test) through Monte Carlo permutations. This method provides an estimation of type I error rate (false positives) by using resampling procedures. Precisely, for each patient and for each electrode showing a significant effect, we computed 20,000 random permutations of the observed trials in two surrogate conditions: trials were randomly assigned to one of the two groups, and then for each permutation, we counted the number of surrogate effects satisfying the observed effect anywhere within a time window of 0–1,000 ms after stimulus onset. Third, statistical values obtained in the Monte Carlo procedure were corrected for multiple comparisons across electrodes using the false discovery rate procedure [91]. The false discovery rate of a test is defined as the expected proportion of false positives among the declared significant results.

### Spectral analyses.

Spectral analysis is exquisitely sensitive to small subcritical epileptic events of small amplitude, which are often hard to detect on conventional voltage time series yet can severely impact on time-frequency diagrams. For spectral analysis, our first stage was therefore a visual inspection of the individual time-frequency diagrams of all electrodes. Artifacts, in the form of large broad-band excursions on occasional trials, were detected in 19 additional electrodes that were therefore eliminated from further analysis. For the remaining 147 electrodes, we used the ‘newtimef' and ‘newcrossf' functions of EEGLAB software [90] to estimate and plot, respectively, the mean event-related (log) spectral perturbation (ERSP) and ITC around word onset. As illustrated in Figures 5 and 6, ERSP and ITC were first calculated separately within each of the four conditions, then the word-present and word-absent conditions within each masking condition were subtracted from each other to yield a time-frequency plot of masked and unmasked effects. We used a prestimulus baseline correction over −400 to 0 ms, and all results are expressed as increases or decreases relative to this baseline (in logarithmic decibel scale for ERSP, in coherence units ranging from 0 to 1 for ITC). Calculation was based on successive windows of 128 samples (= 320 ms) centered on time points −500 to 1,000 ms around stimulus onset. In order to avoid windowing artifacts, we only report results over a window of −400 to 800 ms. We used EEGLAB's default algorithm (fast Fourier transform with Hanning window tapering), a frequency range of 0–100 Hz, and a padding ratio of 1. Prior to computation, individual trials were linearly detrended, and the mean ERP in each condition was removed. Thus, our analysis was aimed at detecting “induced” oscillatory activity, temporally associated with the stimuli but not phase-synchronized with them, while discarding any phase-synchronous “evoked” activity [92–96].

Because of the larger number of available electrodes, times, and frequencies, calculating a corrected-level statistic that evaluates the level of significance of a given time-frequency change in a given electrode poses a severe statistical problem. For simplicity, we therefore only tested significance of effects across groups of electrodes and time-frequency regions of interest. For statistical purposes, we distinguished three time periods (100–200, 200–300, and 300–500 ms relative to word onset) and four frequency bands (alpha = 8–13 Hz; beta = 13–30 Hz; low gamma = 30–50 Hz; and high gamma = 50–100 Hz), thus defining 12 time-frequency regions of interest. For each region, we then computed the mean ERSP for each of the 147 electrodes, and then performed *t*-tests across electrodes to evaluate the significance of the activity induced by masked and unmasked words relative to zero (i.e., relative to prestimulus baseline) and relative to each other. Unless otherwise stated, *p*-values are Bonferroni corrected for the 12 regions tested, and we only report Bonferroni-corrected effects at *p* < 0.05. Identical analyses were performed on mean phase synchrony, except that there were now measures from 1,283 electrode pairs.

### Granger causality.

We adapted a Matlab software package developed by Anil K. Seth [63]. Because our goal was to establish whether relations of causality between electrodes change in the course of the trial, we performed our analyses on successive time windows of 320-ms width, spaced every 25 ms, with Hanning window tapering. For each such window, for each of the four experimental conditions, and for each pair of distinct electrodes (*i*, *j*), the software computed two linear regressions. The first is an autoregressive model that predicts the signal observed on electrode *i* at time *t* based on the signal of the same electrode *i* at previous times *t* − δ*t*, *t* − 2δ*t*,… *t* − *n*δ*t*. The second model is a causal model that adds as regressors to the above regression the signals of the other electrode *j* measured at previous times *t* − δ*t*, *t* − 2δ*t*,… *t* − *n*δ*t*. Here the value of *n* was fixed at eight retrospective samples (= 20-ms retrospective time window) after piloting showed essentially similar results with *n* = 4 or *n* = 16. An associated *F*-value then probes whether the second causal regression accounted for significantly more variance than the first autoregressive model, indicating a putative causal influence of electrode *j* on electrode *i* (see Figure 9 for an example). Seth (2005) suggests using the logarithm of the *F*-test as an index of causality strength, but a problem with this index is that it varies with the number of trials, which differed in the word-present and word-absent conditions [63]. We therefore used a causal index independent of run length: the amount of residual variance that was gained by the second model compared to the first, expressed as a percentage of the residual variance in the second model.

A general finding (see, e.g., Figure 9) was that the presentation of the masks alone already created a considerable increase in Granger causality relative to the baseline. Because we were interested in the further increases due to the presence of the words, over and above the effect of the masks, we calculated a “causal gain” by subtracting the causal index in the word-absent condition from the causal index in the word-present condition.

Finally, note that the causal gain is still a directional measure: there are distinct causal gains for the effects of electrode *j* onto electrode *i* and vice-versa. In the results section, we therefore perform the analysis in two steps: (1) analysis of the mean causal gain, averaged across the two directions of causality, thus probing the existence of causal relations independently of their direction; (2) analysis of the “causal imbalance,” obtained by subtracting the causal gains in the feedforward and feedback directions, thus probing the existence of a preferred direction of causality. For the latter analysis, we defined the feedforward direction as a causal influence of the more posterior electrode onto the more anterior electrode (in case their *y* coordinates were identical, we used the *z* coordinate to define as feedforward an effect of the lowest onto the highest electrode). A positive causal imbalance, therefore, indicated stronger causality in the feedforward or bottom-up direction, and a negative causal imbalance indicated a stronger feedback or top-down causality. Statistical significance was evaluated by *t*-tests over the 1,806 electrode pairs, on causal gains averaged over time windows of 100–200, 200–300, and 300–500 ms. Unless otherwise state, *p*-values are Bonferroni corrected for the three windows tested.

## Supporting Information

Figure S1 Spatial Distribution of Masked and Unmasked Effects Cumulative distributions of ERP effects are plotted against the anterior–posterior axis of Talairach's referential system (*y* coordinate) for the masked (red) and for the unmasked (green) conditions, respectively. The blue curve represents the electrode distribution. Unmasked effects are distributed homogeneously across the electrodes, whereas the distribution of the masked effects is skewed towards posterior electrodes.
(74 KB PPT)

Figure S2 ERSP Effects Separated by Lobes Time-frequency diagrams of mean ERSPs averaged by lobes for masked (left) and unmasked (right) effects. Color indicates log of power variation relative to baseline.
(650 KB PPT)

Figure S3 Granger Causal Imbalance in Two Time Windows Glass brains are superimposed with segments linking, for each patient, all pairs of implanted electrodes. Segments are colored and sized according to the intensity of the feedforward (red) or feedback (blue) causal imbalance, calculated respectively in the 200–300 ms (A) and in the 300–500 ms (B) time windows after word onset. The same scale was used for masked (left column) and unmasked effects (right column).
(714 KB PPT)

Video S1 Absolute Values of iERPs Difference (microVolts) between Word-Present and Word-Absent in the Masked Condition Only electrodes showing a significant effect are displayed as red squares. Square size and color intensity are proportional to the absolute voltage difference between the word and blank conditions. Time ranges from 4 ms after word onset to 799 ms, with 5-ms increments.
(9.38 MB AVI)

Video S2 Absolute Values of iERPs Difference (microVolts) between Word-Present and Word-Absent in the Unmasked Condition Only electrodes showing a significant effect are displayed as red squares. Square size and color intensity are proportional to the absolute voltage difference between the word and blank conditions. Time ranges from 4 ms after word onset to 799 ms, with 5-ms increments.
(9.77 MB AVI)

Video S3 ERSP Difference (dB) in the High-Gamma Band (50–100 Hz) in the Masked Condition Only electrodes showing a significant ERSP increase are displayed as red squares. Square size and color intensity are proportional to the absolute ERSP difference between the word and blank conditions. Time ranges from 2 ms after word onset to 800 ms, with 8-ms increments.
(5.70 MB AVI)

Video S4 ERSP Difference (dB) in the High-Gamma Band (50–100 Hz) in the Unmasked Condition Only electrodes showing a significant ERSP increase are displayed as red squares. Square size and color intensity are proportional to the absolute ERSP difference between the word and blank conditions. Time ranges from 2 ms after word onset to 800 ms, with 8-ms increments.
(5.97 MB AVI)

## Abbreviations

EEG: electroencephalogram
ERP: event-related potential
ERSP: event-related spectral perturbation
iERP: intracranial event-related potential
ITC: intertrial phase coherence
MEG: magnetoencephalography
RT: response time

## References

[pbio-1000061-b001] Del Cul A, Baillet S, Dehaene S (2007). Brain dynamics underlying the nonlinear threshold for access to consciousness. PLoS Biol.

[pbio-1000061-b002] Sergent C, Baillet S, Dehaene S (2005). Timing of the brain events underlying access to consciousness during the attentional blink. Nat Neurosci.

[pbio-1000061-b003] Pins D, Ffytche D (2003). The neural correlates of conscious vision. Cereb Cortex.

[pbio-1000061-b004] Super H, Spekreijse H, Lamme VA (2001). Two distinct modes of sensory processing observed in monkey primary visual cortex (V1). Nat Neurosci.

[pbio-1000061-b005] Lamme VA (2006). Towards a true neural stance on consciousness. Trends Cogn Sci.

[pbio-1000061-b006] Zeki S (2003). The disunity of consciousness. Trends Cogn Sci.

[pbio-1000061-b007] Melloni L, Molina C, Pena M, Torres D, Singer W (2007). Synchronization of neural activity across cortical areas correlates with conscious perception. J Neurosci.

[pbio-1000061-b008] Tong F (2003). Primary visual cortex and visual awareness. Nat Rev Neurosci.

[pbio-1000061-b009] Boehler CN, Schoenfeld MA, Heinze HJ, Hopf JM (2008). Rapid recurrent processing gates awareness in primary visual cortex. Proc Natl Acad Sci U S A.

[pbio-1000061-b010] Koivisto M, Revonsuo A, Lehtonen M (2006). Independence of visual awareness from the scope of attention: an electrophysiological study. Cereb Cortex.

[pbio-1000061-b011] Lamme VA, Roelfsema PR (2000). The distinct modes of vision offered by feedforward and recurrent processing. Trends Neurosci.

[pbio-1000061-b012] Bullier J (2001). Feedback connections and conscious vision. Trends Cogn Sci.

[pbio-1000061-b013] Pascual-Leone A, Walsh V (2001). Fast backprojections from the motion to the primary visual area necessary for visual awareness. Science.

[pbio-1000061-b014] Lamme V (2003). Why attention and awareness are different. Trends Cogn Sci.

[pbio-1000061-b015] Silvanto J, Cowey A, Lavie N, Walsh V (2005). Striate cortex (V1) activity gates awareness of motion. Nat Neurosci.

[pbio-1000061-b016] Rees G, Kreiman G, Koch C (2002). Neural correlates of consciousness in humans. Nat Rev Neurosci.

[pbio-1000061-b017] Vuilleumier P, Sagiv N, Hazeltine E, Poldrack RA, Swick D (2001). Neural fate of seen and unseen faces in visuospatial neglect: a combined event-related functional MRI and event-related potential study. Proc Natl Acad Sci U S A.

[pbio-1000061-b018] Beck DM, Rees G, Frith CD, Lavie N (2001). Neural correlates of change detection and change blindness. Nat Neurosci.

[pbio-1000061-b019] Marois R, Yi DJ, Chun MM (2004). The neural fate of consciously perceived and missed events in the attentional blink. Neuron.

[pbio-1000061-b020] Lumer ED, Rees G (1999). Covariation of activity in visual and prefrontal cortex associated with subjective visual perception. Proc Natl Acad Sci U S A.

[pbio-1000061-b021] Tononi G (2004). An information integration theory of consciousness. BMC Neurosci.

[pbio-1000061-b022] Dehaene S, Naccache L (2001). Towards a cognitive neuroscience of consciousness: basic evidence and a workspace framework. Cognition.

[pbio-1000061-b023] Dehaene S, Changeux JP, Naccache L, Sackur J, Sergent C (2006). Conscious, preconscious, and subliminal processing: a testable taxonomy. Trends Cogn Sci.

[pbio-1000061-b024] Lau HC, Passingham RE (2006). Relative blindsight in normal observers and the neural correlate of visual consciousness. Proc Natl Acad Sci U S A.

[pbio-1000061-b025] Sahraie A, Weiskrantz L, Barbur JL, Simmons A, Williams SCR (1997). Pattern of neuronal activity associated with conscious and unconscious processing of visual signals. Proc Natl Acad Sci U S A.

[pbio-1000061-b026] Lamme V, Zipser K, Spekreijse H (2002). Masking interrupts figure-ground signals in V1. J Cognitive Neurosci.

[pbio-1000061-b027] Tse PU, Martinez-Conde S, Schlegel AA, Macknik SL (2005). Visibility, visual awareness, and visual masking of simple unattended targets are confined to areas in the occipital cortex beyond human V1/V2. Proc Natl Acad Sci U S A.

[pbio-1000061-b028] Ress D, Backus BT, Heeger DJ (2000). Activity in primary visual cortex predicts performance in a visual detection task. Nat Neurosci.

[pbio-1000061-b029] Ress D, Heeger DJ (2003). Neuronal correlates of perception in early visual cortex. Nat Neurosci.

[pbio-1000061-b030] Baars BJ (1989). A cognitive theory of consciousness.

[pbio-1000061-b031] Dehaene S, Kerszberg M, Changeux JP (1998). A neuronal model of a global workspace in effortful cognitive tasks. Proc Natl Acad Sci U S A.

[pbio-1000061-b032] Dehaene S, Sergent C, Changeux JP (2003). A neuronal network model linking subjective reports and objective physiological data during conscious perception. Proc Natl Acad Sci U S A.

[pbio-1000061-b033] Kouider S, Dehaene S (2007). Levels of processing during non-conscious perception: a critical review of visual masking. Philos Trans R Soc Lond B Biol Sci.

[pbio-1000061-b034] Lau HC, Passingham RE (2007). Unconscious activation of the cognitive control system in the human prefrontal cortex. J Neurosci.

[pbio-1000061-b035] Pessiglione M, Schmidt L, Draganski B, Kalisch R, Lau H (2007). How the brain translates money into force: a neuroimaging study of subliminal motivation. Science.

[pbio-1000061-b036] van Gaal S, Ridderinkhof KR, Fahrenfort JJ, Scholte HS, Lamme VA (2008). Frontal cortex mediates unconsciously triggered inhibitory control. J Neurosci.

[pbio-1000061-b037] Dehaene S, Naccache L (2006). Can one suppress subliminal words. Neuron.

[pbio-1000061-b038] Macknik SL, Livingstone MS (1998). Neuronal correlates of visibility and invisibility in the primate visual system. Nat Neurosci.

[pbio-1000061-b039] Rolls ET, Tovee MJ, Panzeri S (1999). The neurophysiology of backward visual masking: information analysis. J Cogn Neurosci.

[pbio-1000061-b040] Kovacs G, Vogels R, Orban GA (1995). Cortical correlate of pattern backward masking. Proc Natl Acad Sci U S A.

[pbio-1000061-b041] Thompson KG, Schall JD (1999). The detection of visual signals by macaque frontal eye field during masking. Nat Neurosci.

[pbio-1000061-b042] Dehaene S, Naccache L, Cohen L, Bihan DL, Mangin JF (2001). Cerebral mechanisms of word masking and unconscious repetition priming. Nat Neurosci.

[pbio-1000061-b043] Naccache L, Gaillard R, Adam C, Hasboun D, Clemenceau S (2005). A direct intracranial record of emotions evoked by subliminal words. Proc Natl Acad Sci U S A.

[pbio-1000061-b044] Fahrenfort JJ, Scholte HS, Lamme VA (2007). Masking disrupts reentrant processing in human visual cortex. J Cogn Neurosci.

[pbio-1000061-b045] Naccache L, Blandin E, Dehaene S (2002). Unconscious masked priming depends on temporal attention. Psychol Sci.

[pbio-1000061-b046] Kiefer M, Brendel D (2006). Attentional modulation of unconscious “automatic” processes: evidence from event-related potentials in a masked priming paradigm. J Cogn Neurosci.

[pbio-1000061-b047] Fabre L, Lemaire P, Grainger J (2007). Attentional modulation of masked repetition and categorical priming in young and older adults. Cognition.

[pbio-1000061-b048] Lachter J, Forster KI, Ruthruff E (2004). Forty-five years after Broadbent (1958): still no identification without attention. Psychol Rev.

[pbio-1000061-b049] Marzouki Y, Grainger J, Theeuwes J (2007). Exogenous spatial cueing modulates subliminal masked priming. Acta Psychol (Amst).

[pbio-1000061-b050] Sumner P, Tsai PC, Yu K, Nachev P (2006). Attentional modulation of sensorimotor processes in the absence of perceptual awareness. Proc Natl Acad Sci U S A.

[pbio-1000061-b051] Nakamura K, Dehaene S, Jobert A, Le Bihan D, Kouider S (2007). Task-specific change of unconscious neural priming in the cerebral language network. Proc Natl Acad Sci U S A.

[pbio-1000061-b052] Kunde W, Kiesel A, Hoffmann J (2003). Conscious control over the content of unconscious cognition. Cognition.

[pbio-1000061-b053] Kentridge RW, Heywood CA, Weiskrantz L (2004). Spatial attention speeds discrimination without awareness in blindsight. Neuropsychologia.

[pbio-1000061-b054] Dehaene S, Changeux JP (2005). Ongoing spontaneous activity controls access to consciousness: a neuronal model for inattentional blindness. PLoS Biol.

[pbio-1000061-b055] Fries P (2005). A mechanism for cognitive dynamics: neuronal communication through neuronal coherence. Trends Cogn Sci.

[pbio-1000061-b056] Buzsaki G (2006). Rhythms of the brain.

[pbio-1000061-b057] Lachaux JP, George N, Tallon-Baudry C, Martinerie J, Hugueville L (2005). The many faces of the gamma band response to complex visual stimuli. Neuroimage.

[pbio-1000061-b058] Tallon-Baudry C, Bertrand O, Fischer C (2001). Oscillatory synchrony between human extrastriate areas during visual short-term memory maintenance. J Neurosci.

[pbio-1000061-b059] Tallon-Baudry C, Mandon S, Freiwald WA, Kreiter AK (2004). Oscillatory synchrony in the monkey temporal lobe correlates with performance in a visual short-term memory task. Cereb Cortex.

[pbio-1000061-b060] Brovelli A, Ding M, Ledberg A, Chen Y, Nakamura R (2004). Beta oscillations in a large-scale sensorimotor cortical network: directional influences revealed by Granger causality. Proc Natl Acad Sci U S A.

[pbio-1000061-b061] Gross J, Schmitz F, Schnitzler I, Kessler K, Shapiro K (2004). Modulation of long-range neural synchrony reflects temporal limitations of visual attention in humans. Proc Natl Acad Sci U S A.

[pbio-1000061-b062] Buschman TJ, Miller EK (2007). Top-down versus bottom-up control of attention in the prefrontal and posterior parietal cortices. Science.

[pbio-1000061-b063] Seth AK (2005). Causal connectivity of evolved neural networks during behavior. Network.

[pbio-1000061-b064] Hesse W, Moller E, Arnold M, Schack B (2003). The use of time-variant EEG Granger causality for inspecting directed interdependencies of neural assemblies. J Neurosci Methods.

[pbio-1000061-b065] Granger CWJ (1969). Investigating causal relations by econometric models and cross-spectral methods. Econometrica.

[pbio-1000061-b066] Quiroga RQ, Reddy L, Kreiman G, Koch C, Fried I (2005). Invariant visual representation by single neurons in the human brain. Nature.

[pbio-1000061-b067] Quiroga RQ, Mukamel R, Isham EA, Malach R, Fried I (2008). Human single-neuron responses at the threshold of conscious recognition. Proc Natl Acad Sci U S A.

[pbio-1000061-b068] Mormann F, Kornblith S, Quiroga RQ, Kraskov A, Cerf M (2008). Latency and selectivity of single neurons indicate hierarchical processing in the human medial temporal lobe. J Neurosci.

[pbio-1000061-b069] Moutoussis K, Zeki S (2002). The relationship between cortical activation and perception investigated with invisible stimuli. Proc Natl Acad Sci U S A.

[pbio-1000061-b070] Farah MJ (2000). The cognitive neuroscience of vision.

[pbio-1000061-b071] Bar M, Tootell RB, Schacter DL, Greve DN, Fischl B (2001). Cortical mechanisms specific to explicit visual object recognition. Neuron.

[pbio-1000061-b072] Grill-Spector K, Kushnir T, Hendler T, Malach R (2000). The dynamics of object-selective activation correlate with recognition performance in humans. Nat Neurosci.

[pbio-1000061-b073] Raymond JE, Shapiro KL, Arnell KM (1992). Temporary suppression of visual processing in an RSVP task: an attentional blink. J Exp Psychol Hum Percept Perform.

[pbio-1000061-b074] Mack A, Rock I (1998). Inattentional blindness.

[pbio-1000061-b075] Allman JM, Hakeem A, Erwin JM, Nimchinsky E, Hof P (2001). The anterior cingulate cortex. The evolution of an interface between emotion and cognition. Ann N Y Acad Sci.

[pbio-1000061-b076] Shanahan M, Baars B (2005). Applying global workspace theory to the frame problem. Cognition.

[pbio-1000061-b077] Logothetis NK (2002). The neural basis of the blood-oxygen-level-dependent functional magnetic resonance imaging signal. Philos Trans R Soc Lond B Biol Sci.

[pbio-1000061-b078] Logothetis NK (2008). What we can do and what we cannot do with fMRI. Nature.

[pbio-1000061-b079] Reddy L, Quiroga RQ, Wilken P, Koch C, Fried I (2006). A single-neuron correlate of change detection and change blindness in the human medial temporal lobe. Curr Biol.

[pbio-1000061-b080] Singer W, Gray CM (1995). Visual feature integration and the temporal correlation hypothesis. Annu Rev Neurosci.

[pbio-1000061-b081] Gray CM, Singer W (1989). Stimulus-specific neuronal oscillations in orientation columns of cat visual cortex. Proc Natl Acad Sci U S A.

[pbio-1000061-b082] von Stein A, Chiang C, Konig P (2000). Top-down processing mediated by interareal synchronization. Proc Natl Acad Sci U S A.

[pbio-1000061-b083] von Stein A, Sarnthein J (2000). Different frequencies for different scales of cortical integration: from local gamma to long range alpha/theta synchronization. Int J Psychophysiol.

[pbio-1000061-b084] Rossetti Y (1998). Implicit short-lived motor representations of space in brain damaged and healthy subjects. Conscious Cogn.

[pbio-1000061-b085] Koch C, Tsuchiya N (2007). Attention and consciousness: two distinct brain processes. Trends Cogn Sci.

[pbio-1000061-b086] Kentridge RW, Heywood CA, Weiskrantz L (1999). Attention without awareness in blindsight. Proc R Soc Lond B Biol Sci.

[pbio-1000061-b087] Husserl E (1992). Méditations cartésiennes: introduction aÌ la pheìnomeìnologie.

[pbio-1000061-b088] Velly LJ, Rey MF, Bruder NJ, Gouvitsos FA, Witjas T (2007). Differential dynamic of action on cortical and subcortical structures of anesthetic agents during induction of anesthesia. Anesthesiology.

[pbio-1000061-b089] Tononi G, Edelman GM (1998). Consciousness and complexity. Science.

[pbio-1000061-b090] Delorme A, Makeig S (2004). EEGLAB: an open source toolbox for analysis of single-trial EEG dynamics including independent component analysis. J Neurosci Methods.

[pbio-1000061-b091] Benjamini Y, Hochberg Y (1995). Controlling the false discovery rate: a practical and powerful approach to multiple testing. J R Statist Soc.

[pbio-1000061-b092] Lachaux JP, Rodriguez E, Martinerie J, Varela FJ (1999). Measuring phase synchrony in brain signals. Hum Brain Mapp.

[pbio-1000061-b093] Mormann F, Lehnertz K, David P, Elger CE (2000). Mean phase coherence as a measure for phase synchronization and its application to the EEG of epilepsy patients. Physica D.

[pbio-1000061-b094] Makeig S, Westerfield M, Jung T, Enghoff S, Townsend J (2002). Dynamic brain sources of visual evoked responses. Science.

[pbio-1000061-b095] Tallon-Baudry C (2003). Oscillatory synchrony and human visual cognition. J Physiol Paris.

[pbio-1000061-b096] Makeig S, Debener S, Onton J, Delorme A (2004). Mining event-related brain dynamics. Trends Cogn Sci.

